# Breaking the cycles of poverty: Strategies, achievements, and lessons learned in Los Cuatro Santos, Nicaragua, 1990–2014

**DOI:** 10.1080/16549716.2017.1272884

**Published:** 2017-01-31

**Authors:** Elmer Zelaya Blandón, Carina Källestål, Rodolfo Peña, Wilton Perez, Staffan Berglund, Mariela Contreras, Lars-Åke Persson

**Affiliations:** ^a^ Asociación para el Desarrollo Económico y Sostenible de El Espino (APRODESE), Chinandega, Nicaragua; ^b^ UNAN-León, León, Nicaragua; ^c^ Department of Women’s and Children’s Health, Uppsala University, Uppsala, Sweden; ^d^ Pan American Health Organization, San Salvador, El Salvador; ^e^ Faculty of Health and Society, Malmö University, Malmö, Sweden; ^f^ Department of Infectious Disease Epidemiology, London School of Hygiene & Tropical Medicine, London, UK

**Keywords:** Poverty alleviation, community participation, microcredits, education, water and sanitation, scale-up, under-five mortality, Health and Demographic Surveillance System

## Abstract

**Background:** In a post-war frontier area in north-western Nicaragua that was severely hit by Hurricane Mitch in 1998, local stakeholders embarked on and facilitated multi-dimensional development initiatives to break the cycles of poverty.

**Objective:** The aim of this paper is to describe the process of priority-setting, and the strategies, guiding principles, activities, achievements, and lessons learned in these local development efforts from 1990 to 2014 in the Cuatro Santos area, Nicaragua.

**Methods:** Data were derived from project records and a Health and Demographic Surveillance System that was initiated in 2004. The area had 25,893 inhabitants living in 5,966 households in 2014.

**Results:** A participatory process with local stakeholders and community representatives resulted in a long-term strategic plan. Guiding principles were local ownership, political reconciliation, consensus decision-making, social and gender equity, an environmental and public health perspective, and sustainability. Local data were used in workshops with communities to re-prioritise and formulate new goals. The interventions included water and sanitation, house construction, microcredits, environmental protection, school breakfasts, technical training, university scholarships, home gardening, breastfeeding promotion, and maternity waiting homes. During the last decade, the proportion of individuals living in poverty was reduced from 79 to 47%. Primary school enrolment increased from 70 to 98% after the start of the school breakfast program. Under-five mortality was around 50 per 1,000 live births in 1990 and again peaked after Hurricane Mitch and was approaching 20 per 1,000 in 2014. Several of the interventions have been scaled up as national programs.

**Conclusions:** The lessons learned from the Cuatro Santos initiative underline the importance of a bottom-up approach and local ownership of the development process, the value of local data for monitoring and evaluation, and the need for multi-dimensional local interventions to break the cycles of poverty and gain better health and welfare.

## Background

### Global goals and local processes

At the completion of the Millennium Development Goal (MDG) era (1990–2015), reports have assessed progress made and challenges that remain [1–3]. Globally, the number of people living in extreme poverty has been halved, the number of out-of-school children has also been reduced by half, gender equality in education has progressed markedly, and under-five mortality has dropped from 90 per 1,000 live births in 1990 to 43 per 1,000 in 2015 [4,5]. Still, there are inequities in these achievements across and within countries [6]. In most cases only global or national perspectives on progress in development indicators have been provided [2,7], while the local processes that underpin the national and global results have rarely been displayed [8–10].

### Top-down versus bottom-up perspectives

Frequently a top-down view is provided on health and development [3,11]. More rarely a bottom-up perspective is employed with local participatory approaches [12,13]. The different MDG-related interventions have usually focused on one particular goal, for example, the reduction of child mortality. The Sustainable Development Goals imply a paradigm shift to a multi-dimensional perspective on development [14], health, and welfare [15] that also includes an environmental point of view [16].

### Nicaraguan achievements

Nicaragua has been relatively successful in reaching the MDGs. At the end of the MDG period, there was a reduction of three-quarters in poverty, and hunger decreased by two-thirds [17]. Enrolment in primary education had become high and gender-equitable. The decline in child mortality was 65%, and maternal mortality was at a moderate level. These achievements should also be viewed against the background of the hardship of the Nicaraguan revolution and the Contras war of the 1980s [18]. In spite of those difficulties, that early period also implied considerable progress in literacy and education [19,20], improved child survival, reduced total fertility rates [21–24], and advances in poverty alleviation [25,26]. The Nicaraguan communities have been involved in a broad range of development activities, for example, the national literacy campaign, inspired by the pedagogy of Paulo Freire [20,27].

### The Cuatro Santos example

Los Cuatro Santos is a frontier area in north-western Nicaragua that was profoundly affected by the war in the 1980s and by Hurricane Mitch in the 1990s. The population in this area has been successful in poverty alleviation [26] and reduction of under-five mortality [24]. The area has also been studied with a focus on the nutrition transition, infant and child feeding and nutritional status [28–30], migration and remittances [31], and environmental improvements [32]. This area provides an opportunity to study whether it is possible in a post-war and post-catastrophe setting to break the cycles of poverty. The aim of this paper is to document and analyse the process of priority-setting, as well as the strategies, guiding principles, activities, achievements, and lessons learned in these local development efforts from 1990 to 2014 in Los Cuatro Santos area, Nicaragua.

## Methods

This paper has a narrative element, which describes the process of the development activities and analyses the achievements and lessons learned over two decades in a post-conflict and post-catastrophe area. The descriptive data were based on a Health and Demographic Surveillance System (HDSS) that provides population-based information on demographic, socio-economic, and selected health indicators over time.

### Setting

Los Cuatro Santos (four saints) area is situated in the northern part of Chinandega, Nicaragua, and is composed of four similar-sized municipalities: San Juan de Cinco Pinos, San Pedro del Norte, Santo Tomas del Nance, and San Francisco del Norte. The whole area covers 310 km^2^ with a population of 25,893 inhabitants in 5,966 households (2014). It is located 250 km northwest of the capital Managua in a mountainous area along the border with Honduras. This region suffered from the war in the 1980s and was seriously affected by Hurricane Mitch in October 1998. Cultivation of basic grains and livestock, which was the traditional source of income, has recently been complemented by an increasing number of small service enterprises. In the last decade, a significant proportion of the population has emigrated to neighbouring countries due to the difficult economic situation and the high rates of unemployment [31].

### Data sources

A wide range of project-related documents dated from 1996 to 2015 served as a basis for the description of the process over time. These included proposals, reports, local official documents, and a strategic 15-year plan that was jointly developed in 2002. The long-term plan was prepared in a facilitated workshop with representatives from the local NGOs, the population movement *Movimiento Comunal*, key persons within the civil society, the mayors of the municipalities, and representatives from the local ministries of Health and Education.

An HDSS was initiated in 2004 in Los Cuatro Santos area covering the whole population and gathering updated demographic and selected health information. Data were collected in a series of surveys linked by household and individual information. Demographic changes (birth, death, migration) between the surveys were carefully registered and dated. The first survey in 2004 had follow-ups in 2007, 2009, and 2014. Household data included information on the house (floor, walls) and services (water, sanitation, electricity) [26]. Unique identifiers of households and individuals linked the databases. The demographic information was collected from all members of the family. All women aged 15–49 years living in the households provided retrospective reproductive histories. Births, deaths, causes of child death by verbal autopsy, and in- and out-migration were registered [24]. The geographic location of every household was mapped by a geographic positioning system (GPS) technology. Additionally, the information covered child nutrition (weight, height), food security, and participation in interventions. Fieldwork was carefully supervised, forms were checked before computerisation, and the forms were returned to the field if the information was missing or suspected to be incorrect. Further quality controls were completed after computerisation including logical controls. Data were carefully cleaned and stored in relational databases. Reports were created after completion of the different follow-up surveys.

### Definitions and analyses

The project-related documents were used to validate the narrative part of this paper. The descriptive quantitative information was based on the HDSS databases. Household size was defined as the number of persons residing in the household at the time of the field survey. Migration was defined as household members aged 18–65 who migrated for economic reasons during the study period to a place outside the study area (including fixed or seasonal work or search for employment). Poverty was measured using the Unsatisfied Basic Needs Index [33,34]. This index is composed of four components: (1) information on housing conditions (unsatisfied: walls of wood, cartons, or plastic and earthen floor); (2) access to water and latrine (unsatisfied: water from river, well, or bought in barrels and no latrine); (3) school enrolment of children (unsatisfied: children 7–14 years of age were not attending school); and (4) education of head of the family and dependency ratio (unsatisfied: head of the family illiterate or dropped out of primary school and dependency ratio > 2.0). Each component received a score of one if unsatisfied. The total sum varied from zero to four. Non-poor households were those with zero or one unsatisfied basic need, poor households had two to three unsatisfied basic needs, and extremely poor households had four unsatisfied basic needs. Poverty transition was defined as the change from one state of poverty in a given year to another poverty status in a different year. The dependency ratio was calculated as the number of people in the family who were aged < 15 or > 65, divided by the number of people between 15 and 65. The total fertility rate was calculated as the sum of age-specific fertility rates in 5-year intervals between ages 15–49 years in a given calendar year. The under-five mortality rate was calculated as the total number of deaths of children aged 0–59 months divided by the total number of live births in a given calendar year. The neonatal mortality rate was calculated as the total number of deaths of infants aged 0–28 days divided by the total number of live births in a given year. For the purpose of graphical display of the mortality rates, 3-year moving averages were used to smooth the curve. A GPS-based map of household poverty was produced to illustrate the usefulness of spatial information.

## Results

The population in Los Cuatro Santos increased by an estimated 25% in the early 1990s with the return from Honduras of families who had fled the fighting and hardship in the 1980s. This influx of people also included previous Contras fighters. In 2004 there were 4,451 houses and 24,095 inhabitants, increasing to 5,966 houses and 25,893 inhabitants in 2014 (Table 1). Only 16 households refused to participate in the HDSS.

**Table 1.** Social and demographic characteristics of households and inhabitants in the Cuatro Santos area, Nicaragua, in 2004 and 2014.

Characteristic	Level	2004	2014
*Households*		*n *= 4,484	*n *= 5,959
Walls	Cement	881/4,484 (19.6%)	1,714/5,959 (28.7%)
	Rammed earth	3,603/4,484 (80.4%)	4,245/5,959 (71.2%)
Floor	Cement	1,133/4,484 (25.3%)	2,622/5,959 (44.0%)
	Dirt floor	3,351/4,484 (74.7%)	3,337/5,959 (55.9%)
Water source	Pipe	1,070/4,484 (23.8%)	2,158/5,959 (36.2%)
	Owned well	2,290/4,484 (51.1%)	3,016/5,959 (50.6%)
	River	1,124/4,484 (25.1%)	785/5,959 (13.1%)
Sanitation	Latrine	3,291/4,484 (73.4%)	4,710/5,959 (79.0%)
	No latrine	1,193/4,484 (26.6%)	1,249/5,959 (20.9%)
Poverty	Non-poor	929/4,484 (20.7%)	3,350/5,959 (56.2%)
	Poor	2,685/4,484 (59.8%)	2,575/5,959 (43.2%)
	Extremely poor	837/4,484 (18.6%)	34/5,959 (0.6%)
*Population*		*n = 23,265*	*n = 25,946*
Age (years)	< 5	2,822/23,265 (12.1%)	2,622/25,946 (10.1%)
	5–14	7,090/23,265 (30.5%)	5,630/25,946 (21.6%)
	15–64	12,180/23,265 (52.4%)	16,043/25,946 (61.8%)
	≥ 65	1,173/23,265 (5.0%)	1,651/25,946 (6.3%)
Education (age 15 years or more)	Illiterate	2,461/13,353 (18.4%)	1,959/17,694 (11.1%)
	Lower primary	5,444/13,353 (40.8%)	6,007/17,694 (33.9%)
	Primary completed	1,995/13,353 (14.9%)	2,763/17,694 (15.6%)
	Lower secondary	1,963/13,353 (14.7%)	3,106/17,694 (17.6%)
	Secondary completed	840/13,353 (6.3%)	2,709/17,694 (15.3%)
	Lower university	151/13,353 (1.1%)	601/17,694 (3.4%)
	University completed	344/13,353 (2.6%)	459/17,694 (2.6%)
	Other	155/13,353 (1.2%)	90/17,694 (0.5%)

Note: Data are *n* or *n/n* (%).

### Popular participation

In 1996, a local group of people in Los Cuatro Santos took the initiative to discuss possible local development initiatives, inspired by the experiences of the Nicaraguan literacy campaign [20] and based on the pedagogies of Paulo Freire [27]. This group further developed into a local Non-Governmental Organisation (NGO) that was labelled Asociación para el Desarrollo Económico y Sostenible de El Espino (APRODESE), referring to the local village where the first development activities were initiated. This NGO has since then had a major role, together and in collaboration with other local NGOs and the local government, in promoting the development efforts in the area. In these discussions and later planning, the approach to ‘decode reality’ was applied (Box 1). This methodology included an analysis of the past and present situation, provided a theoretical input on possible solutions, and produced a wish list that was narrowed down to a shortlist of priority actions. This approach implies local ownership of decisions and actions taken. The first prioritised activities included water and sanitation, microcredits, home gardening, environmental protection, technical training of young people, and initiation of a solar-powered radio-link network for voice communication and Internet access (Table 2).

**Table 2.** Prioritised activities, rationale, problems encountered, and achievements in the Cuatro Santos area, Nicaragua.

Activities	Year of start	Rationale	Problems encountered	Achievements
Microcredits	1997	Development of local economy	History of no payback behaviour	Development of a local microcredits scheme via a professional NGO bank. Payback > 95%
Home gardening	1997	Increase local food production	Lack of water	800 families with diversified home gardens, drip irrigation
Environmental protection	1997	Stop deforestation and protect soil and basins	Resistance to change practices such as forest fires	No more massive forest fires in the area. More than 200,000 trees planted. 50,000 m of stone walls, 80,000 m of ditches
Technical training	1997	Promote youth employment and entrepreneurship	Low women’s participation in traditional men’s professions	2,122 boys and girls intensively trained and certified
Information,communication	1997	No telephone system, no access to Internet	Lack of electricity, resistance of companies to delivering IT services, high price of solar energy	Solar-powered radio-link network established including Internet, cyber cafés, > 2000 children trained in computer use and Internet
House construction	1998	Replace hurricane-destroyed houses	Resistance of people to moving to safer areas	300 new houses constructed
Piped water	1999	Poor water quantity and quality	Scarcity of ground water, no electricity in some areas	1,300 new houses with piped water including water meters
School breakfast	1999	Increase school coverage and performance	Water quality; led to drinking water projects at schools	Primary school attendance increased from 70 to 98%. Scaled up as national policy to all public schools in 2007
University scholarships	2000	Strengthening local professional capacities	Low quality of some university training. Some dropouts	More than 185 new professionals in the area in different specialties. León University opens local branch
Maternity waiting homes	2005	Promote institutional delivery	Lack of food at home for other children	Increase of institutional deliveries from 52% in 2004 to 90% in 2014. Food packs for waiting families at home

### Hurricane Mitch

In October 1998, the area was hit by Hurricane Mitch with the destruction of houses, water and sanitation installations, gardens, cultivated fields and forests, roads, and bridges [35]. The locally formed development group got involved in the immediate emergency responses to provide food, shelter, and medical assistance.

### Long-term planning

In 2002, based on the same ‘decoding reality’ strategy a long-term strategic plan up to 2015 was developed by local stakeholders, and community and NGO representatives. A set of values and guiding principles constituted a basis for the choices made and the strategies employed. These included local and joint ownership of the activities (bottom-up local collaborative effort), political reconciliation (Sandinistas and ex-Contras working together), consensus decision-making (part of the ‘decoding reality’ approach), social and gender equity (stressed in the implementation and evaluation of activities), an environmental and public health perspective (aiming at better welfare), and sustainability (economically and regarding continued effects of investments and actions). The challenges of the post-catastrophe situation were met with continued and expanded development activities (Table 2).

Leaders of the local NGOs, who were trained in the Nicaraguan literacy campaign [20], usually acted as facilitators. The concepts of local participation and ownership were central, and processes of reconciliation and consensus decision-making were initiated in case of conflicts. Voluntary work was invested; for example, village inhabitants dug an estimated 40 km of ditches for the piped water installations, and students with university scholarships devoted part of their free time to community work.

### Safe water

**Figure 1.**
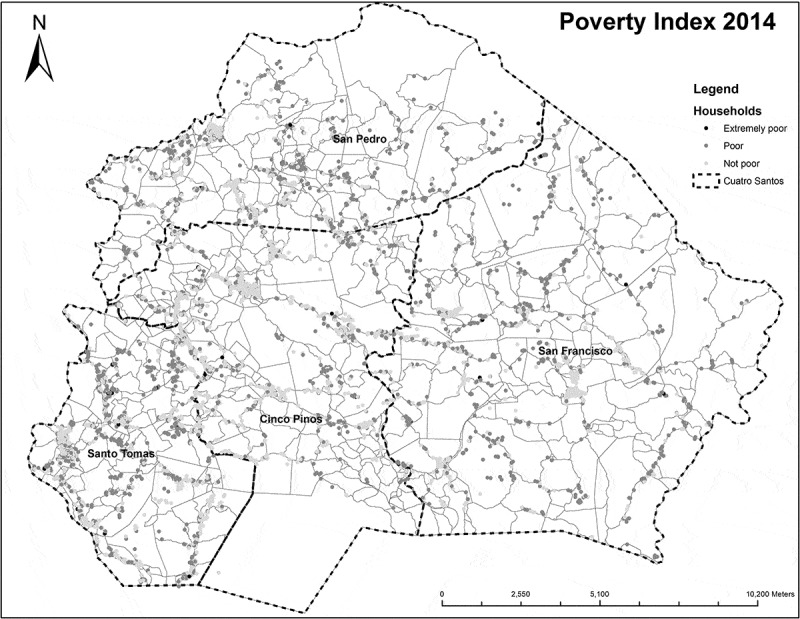
The Cuatro Santos area, Nicaragua. Households in 2014 marked and classified as not poor, poor, or extremely poor based on the Unsatisfied Basic Needs Index.

Safe drinking water was provided by piped water systems. A significant experience was that water meters were needed for appropriate billing, to reduce the excessive use of water and promote the sustainability of the system. Due to the need of electricity for pumps, there was an unequal distribution of piped water systems along the roads that excluded poorer households in remote areas from this resource. HDSS data and maps were used in workshops to discuss a new direction for the piped water efforts (Figure 1). Recently communities located more than 5 km from the main road have been prioritised and water has been supplied by the use of gravity or mechanical pumps. The water program was also used as an entry point for a household’s participation in other activities, for example, the technical training of young people, and microcredits as well as health education (sexual health, HIV prevention, and maternal and child health). The use of water meters has now been implemented in piped water systems across the whole country.

### Microcredits

The microcredits were initiated just before the hurricane catastrophe. Two NGO microfinance banks provided loans that varied from a few hundred to 5,000 US dollars. The primary purpose was to finance agricultural and small industrial activities. About US $5 million has been invested in microcredit programs in Cuatro Santos, and the majority of loan-takers were women. At an early stage, the importance of complete payback of loans was evident, which has been crucial for sustained microcredit activities. Links between different activities were created; for example, one’s participation in the microcredit program was conditional upon sending all one’s children to primary school.

### Home gardening

Home gardening was prioritised in the area and implemented through a participatory process of farm planning that followed the ‘decoding reality’ approach. The main products for cultivation were vegetables, fruits, cassava, maize, and beans. Most of the harvest was for the farmers’ consumption, but some could be sold to pay for other food, school uniforms, and supplies for the children. There was a lack of water in the area, a problem that was partly managed by drip irrigation. The home gardening activities were used as an entry point for gender sensitisation activities.

### Environmental protection

To stop deforestation and forest fires and to protect soils and basins, a program was initiated that included advocacy against forest fires, tree plantation in forests, as well as fruit-tree plantations in the gardens. These activities stopped the forest fires in the area. Stone walls and ditches were constructed and dug to protect the soil and the water basins.

### Technical training

At an early stage in the process (1998), an educational centre was built in Cuatro Santos to offer professional training courses free of charge to young people in the area. This education program was certified by the national authority, INATEC (The National Technical Institute), and has so far (2015) trained 2,122 young women and men in carpentry, house construction, handicraft, welding, electricity, sewing, computer science, baking, cooking, agriculture, animal health, and solar energy techniques. Students who graduated were also given the possibility of obtaining a small amount of credit to procure essential equipment and start their own businesses, and the best students were given scholarships for further studies at the university.

### Information and communication technologies

In the initial planning workshops, the lack of telephones and access to the Internet was prioritised. A solar panel-supported radio-link network was established and proved to be of great importance in the post-catastrophe operations. This system was expanded and improved, and an Internet café was set up at the training centre. Most school-age children in the area received a one-day training session in computer and Internet use, increasing the computer literacy of the young generation in the area from zero to almost complete.

### School breakfasts and school attendance

School breakfasts were implemented to attract children to enter and complete primary education. In the pilot school, initially, 30% of eligible children were not attending primary school and 30% of the attending children had not eaten breakfast. After 3 weeks, all eligible children attended school. A glass of locally produced fruit juice and a nutrient-enriched biscuit were provided to each child every morning. The program was an immediate success in attracting earlier school dropouts and stimulating families to send their children to the primary schooling level. Primary school attendance in the area is now 98%. The program was initiated in a village school in Cuatro Santos and concentrically expanded to all schools in the area. Based on this local experience, the Ministry of Education has now scaled up this program to all public schools in Nicaragua.

### University scholarships

A large number of young adults have received scholarships for university training in a variety of fields that were prioritised based on local needs and interests shown. The students were, for example, trained in agriculture, medicine, computer science, administration, accounting, mechanics, electronics, and nursing. Many are now working in local governments and institutions in the area. Based on these positive experiences, the local NGOs and stakeholders succeeded in convincing the León University authorities to open a local university branch in Somotillo, the closest larger municipality.

### Health education and maternity waiting homes

Health education has always been a component of the activities of the local NGOs. The HDSS data were also used to produce pamphlets that included local data and basic health messages, for example, in relation to infant feeding and the management of common childhood illnesses. The HDSS information revealed that some women, especially those living in remote areas, delivered at home due to distance and lack of transport. This knowledge made the local stakeholders prioritise maternity waiting homes, where expectant women could stay nearer to the delivery unit of the health centre and have access to skilled attendance at delivery. Some women could not use this resource, for example, if the rest of the family were not able to get food at home during the days spent at the waiting home. This problem was counteracted by a food package activity, which was successfully offered to those families. The maternity waiting homes have now been implemented in prioritised areas across the whole country based on the experiences of those living in Los Cuatro Santos area.

### Cook stoves, roads

The local HDSS data revealed that 89% of the households had cook stoves without a chimney, usually inside the houses. These stoves contributed to indoor air pollution and most likely to an increased risk of respiratory tract infections [36]. A pilot model of an energy-efficient, insulated cook stove with a chimney (named Crucita) was developed and technically evaluated [37]. Some cook stoves have been constructed in the area as part of this pilot, and plans are underway to scale up this innovation to improve energy efficiency, reduce the amount of firewood used, and improve the environment for the household members (temperature, air pollution). The local NGOs and stakeholders also successfully convinced the government to construct a new tarmac road through the area replacing the old rough road in 2006–2010. The new road has improved travel and trade in the area significantly.

### Local data for planning and priority-setting

The population data on demographic, socio-economic, and health characteristics that became available from 2004 with repeated updates have served multiple purposes. They have been used for planning, prioritisation, and evaluation purposes in workshops with local stakeholders and communities. Inequities in infrastructure, for example, water and sanitation, or in the utilisation of services, such as delivery at health institutions, became evident and provoked action when facts were displayed and discussed. Maps based on GPS data on important outcomes, for example, the level of poverty, were made available for discussion with local politicians and stakeholders (Figure 1). The HDSS updates were summarised in reports that also included census data for each municipality. Local politicians have used this information in their efforts to get access to resources from the national level. Communities also received feedback from their data through leaflets that included relevant facts and important health messages.

### Demographic and health indicators

**Figure 2.**
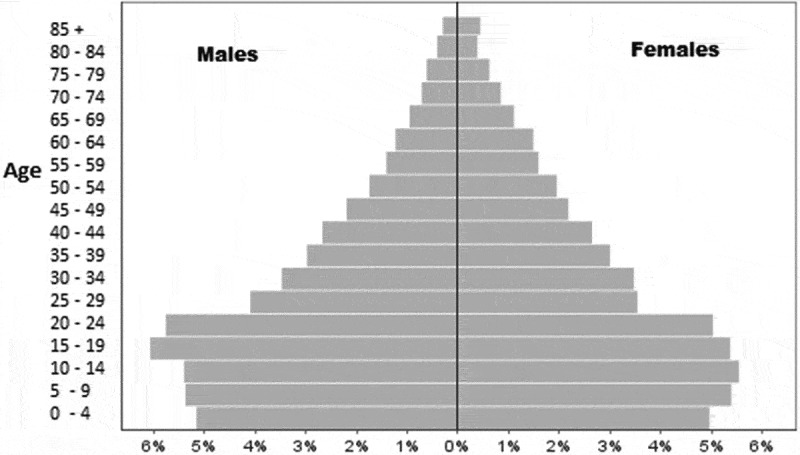
Population pyramid of the Cuatro Santos area, 2014. Total population 25,893 inhabitants.

**Figure 3.**
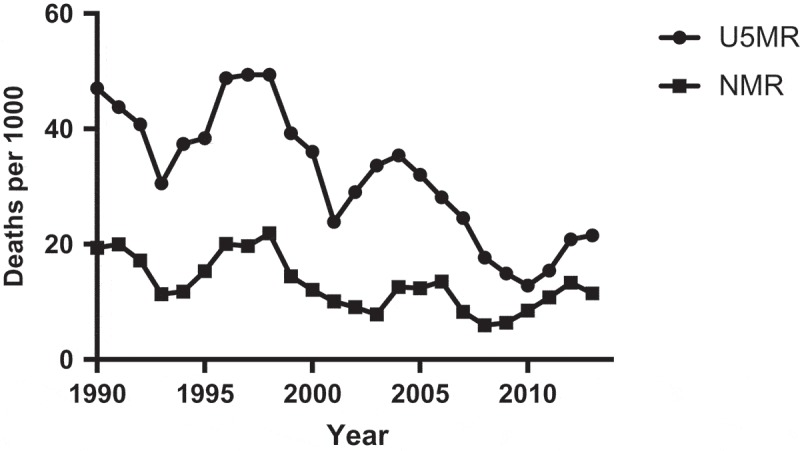
Mortality in children below the age of 5 years (U5MR, deaths per 1,000 live births) and neonatal mortality (NMR, deaths ≤ 28 days of age per 1,000 live births) 1990–2014 in the Cuatro Santos area, Nicaragua. Total number of live births 15,740.

There has been a substantial change during the last decade (2004–2014) in several demographic, economic, and health indicators. Poverty, expressed as unsatisfied basic needs, decreased from 79 to 47%, and extreme poverty was almost not present at the end of the study period (Table 1). A gradual increase in the size of the population was accompanied by a decrease in the number of children below 5 years of age. The total fertility rate decreased from 3.12 in 2004 (95% CI 2.95–3.28) to 2.51 in 2014 (95% CI 2.38–2.64). The dependency ratio decreased from 0.91 to 0.62. These patterns were also shown in the population pyramid of 2014 that reflects the previous higher birth rates, the later lower total fertility rate, and the increasing child survival (Figure 2). Booking for antenatal care was 97% in 2004 and 98% in 2014. Institutional deliveries increased from 54% in 2004 to 94% in 2014. The mortality rate of children below the age of 5 years (U5MR), which was around 50 per 1,000 live births in 1990, was reduced in the following years but increased again at the time of Hurricane Mitch (Figure 3). In 2010, the U5MR was around 15/1,000 followed by an increase towards the end of the study period to approximately 20 per 1,000 live births. The neonatal mortality (NMR) has, with some variation, persisted the whole time and constituted an increasing proportion of the U5MR.

## Discussion

### Lessons learned

We have described a development process from 1990 to 2015 in a post-war frontier area in Nicaragua that was severely hit by Hurricane Mitch in 1998. Through a wide range of facilitated community-based activities improvements were made in the natural environment and infrastructure, and to health and well-being. The lessons learned underline the importance of a bottom-up approach and local ownership of the development process, the importance of the collection of local data for monitoring and evaluation, and the importance of multi-dimensional local interventions to break the cycles of poverty.

### The research and development process and transferability of findings

This paper mainly uses a narrative approach to describe the development process, supported by population-based data of an HDSS that were available from 2004 onwards. The data cover the whole population. Non-participation in the surveillance due to refusal was very limited. Fieldwork with trained local data collectors was always supervised, and any errors were corrected. The first author has been the leading actor in the development activities from the start and also had the primary responsibility for organising the HDSS field activities. The activities have not included any randomised interventions. It is plausible that the rapid development in the area, for example, regarding poverty alleviation and child survival, is a result of the multi-dimensional local interventions. Conventional epidemiological and statistical techniques may not be suitable to analyse these complex associations. Data mining approaches may be used to explore and discover patterns of interactions between interventions on selected outcomes and how these are modified by contextual factors [38]. That is, however, outside the scope of this paper. The transferability of findings is mainly related to the experiences of the strategies employed and the guiding principles, as problems and priorities are local and the local context influences the development processes. The transferability of findings might also be illustrated by the national scale-up of some of the different activities. The national implementation of school breakfasts was based on the positive outcomes of school breakfasts for primary education attendance in Cuatro Santos. The usefulness of maternity waiting homes was also appreciated and implemented at national level in areas where distances and lack of transport forced women to deliver at home without access to skilled attendance. Relevant details, such as the necessity of water meters at each household for sustained piped water installation, have also become national policy.

### Ascendance out of poverty

From 2004 to 2014 a lot of individuals in the area ascended out of poverty, and extreme poverty (based on the Unsatisfied Basic Needs Index) was almost eradicated. On the national level, Nicaragua has managed to reduce the proportion of individuals living in extreme poverty (living on less than 1.25 dollars per day) from 32.7% in 1993 to 8.5% in 2009 [17]. In an earlier paper from our group, we showed that participation in a microcredit program, the involvement of young individuals in technical training, and having households practise home gardening were all associated with the ascent out of poverty [26]. Migration out of or within the country often takes place to improve living conditions and access to welfare services, for example, health care. This drive for improvement was also the case in the Cuatro Santos area, as shown in a recent doctoral thesis [31]. Although remittances have contributed to wealth in the area, the net gain in relation to poverty reduction is difficult to assess, as the out-migration also implies a loss of local productivity.

### Assessing poverty

The Unsatisfied Basic Needs assessment has been used to monitor households and communities over time and for comparisons between geographical areas [33,34]. A wealth index, for example, an asset score, is an alternative way of classifying household assets and classifies a population on a poor–rich scale [39,40]. This index has been widely used in large surveys, such as the Demographic Health Survey [40], and serves as a useful tool in classifying other outcomes along a wealth axis. A disadvantage is that this tool cannot be used to follow or compare development over time because each index is only accurate for the survey for which it was created.

### The food security situation

It should be noted that the Unsatisfied Basic Needs assessment does not capture the food security situation. In 2009, we assessed the food security situation in all households in the area with a child below 3 years old. Only 6% of the households had a secure food situation the previous month, and 36% had experienced a severe food insecure situation, according to the Household Food Insecurity Access Scale [29]. Our studies with a nutrition focus have shown that this rural society is currently undergoing a nutrition transition [28]. There was a low prevalence of exclusive breastfeeding combined with a suboptimal complementary diet. Consumption of high-energy-density snacks and sugar-sweetened beverages was observed at an early age. Stunted growth of children co-existed with overweight children. Mothers with the lowest level of education were more likely to be exclusively breastfeeding their infants, while they also had lower dietary diversity. Children residing in households with the highest food insecurity were more prone to have an inadequate dietary diversity [29].

### Synergies

It is important to recognise that there are vital synergies between different aspects of human development. Poverty reduction, investments in education, and increased accessibility to potable water may have synergistic effects and not only influence progress in the related indicators but also have measurable effects elsewhere, for example, on mortality of children. Most scientific reports and discussions focus on the effect of single medical interventions on under-five mortality and rarely on the potential impacts and synergies of non-medical, community developmental interventions. However, a recent overview of systematic reviews on interventions for sustainable development and health indicates that investments in the different Sustainable Development Goals are associated with improvements in health [41].

The community engagement for development in this area started as a response to the devastating effects of Hurricane Mitch in 1998. As a consequence of climate change the frequency of extreme weather and climate-related catastrophes will increase [42]. The local stakeholders in Los Cuatro Santos were aware of these challenges. In the home gardening intervention the lack of water was counteracted by drip irrigation. They initiated different environmental protection activities to stop deforestation and protect soils and basins. The technical training had an environmental profile with solar energy techniques incorporated. The innovative cook stoves reduced the use of firewood and improved the home environment. Health in the Anthropocene epoch (when human activities have significantly impacted the Earth’s ecosystem) opens up a new paradigm, where our present needs must safeguard the Earth’s life-support system that decides the welfare of future generations [16]. The Sustainable Development Goal agenda integrates the poverty, health, and welfare perspectives with the need to counteract climate change.

The under-five mortality rate, which was around 50/1,000 live births in 1990 and peaked at the time of Hurricane Mitch in 1998, was below 15/1,000 in 2010 and well on the way to being in line with the goal of the fourth MDG, that is to say, a two-thirds reduction in under-five mortality. Neonatal mortality has been relatively persistent over the years, and over the last 5 years there has been a tendency towards an increase in neonatal mortality in spite of a high proportion of deliveries being performed at health institutions. This phenomenon, which also has been described in other settings, may be linked to insufficient quality of delivery services and newborn care within the health system [43].

### Cuatro Santos as a ‘model’ example

We need to be inspired by good examples of local development initiatives in the continued efforts to reach the Sustainable Development Goals. During the MDG era, the establishment of ‘model villages’ was a way of encouraging development in low-resource settings. The Millennium Villages Project implemented a broad package of community-based interventions to promote economic development in 80 poor rural sites from 10 African countries. Early results were encouraging [44], but the project was critiqued foremost for the top-down design, the supposed weak sustainability, and the overrated effects on outcomes such as under-five mortality [45,46].

## Conclusions

The achievements and lessons learned from the Cuatro Santos initiative illustrate the importance of a bottom-up approach and local ownership of the development process, the importance of local data for monitoring and evaluation, and the importance of multi-dimensional interventions to ascend out of poverty and gain better health and welfare.

**Box 1.** The ‘decoding reality’ approach employed in the development work in Los Cuatro Santos area, Nicaragua.

1. Reviewing the past and present	It allows the workshop participants to look back on their past situation, identifying main achievements and problems encountered. Questions: how was the situation? What were the main problems? With which resources did we work on solving these problems? When did that happen? Where? (Current action)
2. Theoretical and technical input	A theoretical input is provided, pointing at evidence-based solutions to (some of) the problems identified. This may lead to a proactive attitude of the participants. (Reflexion)
3. Wish list	Based on the analysis of the past and present situation and the theoretical input, the participants are asked to provide a list of possible actions (maybe 20) to improve their lives. (New action)
4. Shortlisting prioritised possible actions	A selection of short-term doable lists of activities (maybe three) is made with defined responsibilities and deadlines for each participant.

## Paper context

In the development discussion a top-down view is frequently provided. The local processes that underpin global results are rarely displayed. This report from a frontier area in Nicaragua describes how local stakeholders took development initiatives to ascend out of poverty. The characteristics included local ownership, bottom-up approaches, and multi-dimensional interventions. Local data reinforced the process. The lessons learned may serve as a local example of the continued efforts to reach the UN Sustainable Development Goals.
